# Cell Population Dynamics Informed by Cell-Cycle Regulation: A Deterministic Modeling Toolkit

**DOI:** 10.34133/csbj.0192

**Published:** 2026-08-14

**Authors:** Elsi Ferro, Antonio Laus, Rossano Atzeni, Maria Valentini, Enrico Pieroni, Massimo Pisu

**Affiliations:** CRS4 (Center for Advanced Studies, Research and Development in Sardinia), Pula, Italy.

## Abstract

Cell populations grow, shrink, and reshuffle their composition as individual cells divide, arrest, and die. Because cell division is paced by progression through the cell cycle, cell-cycle control is the fundamental axis linking intracellular regulation to population-level dynamics. Quantitatively capturing how cell-cycle regulation affects population dynamics is thus central to understanding tissue homeostasis, tumor expansion, immune responses, and the performance of cell-based bioprocesses—from tissue engineering and regenerative applications to the manufacturing of vaccines and recombinant therapeutics. In this view, a broad spectrum of mathematical models has been proposed to describe cell population dynamics, ranging from phenomenological growth laws that treat net proliferation as a black box to structured descriptions that resolve single-cell heterogeneity and link population change to cell-cycle progression. In this review, we survey deterministic frameworks for modeling cell-cycle-informed population dynamics, highlighting how modeling choices map onto accessible experimental readouts and biomedical and biotechnological questions. In doing so, we aim to provide a theoretical roadmap for readers new to the field who seek to translate intracellular cell-cycle regulation into empirically grounded population-level models. We organize models along 2 conceptual dimensions: the representation of cell-to-cell heterogeneity through increasing levels of population structure and the level of mechanistic specification of cell division through cell-cycle regulation. In particular, we discuss practical approaches to couple cell-cycle regulation to population-level dynamics—from coarse-grained to explicit multiscale couplings—and how external perturbations enter such couplings. We conclude by outlining open challenges toward cell-cycle-aware population models that match mechanistic resolution with experimental identifiability.

## Introduction

Life unfolds through the continuous appearance and disappearance of cells. New cells are born through cell division—paced by progression through the cell cycle—and are lost through cell death—limiting their indefinite accumulation.

Understanding how cell populations evolve is therefore essential both for interpreting experimental observations and for designing interventions that shift this balance in a controlled way, from inhibiting tumor growth to increasing yields in bioprocessing systems.

Mechanisms shaping cell population dynamics span a variety of intracellular and extracellular processes, including metabolic and nutrient-sensing programs, growth-factor signaling, cellular stress responses, DNA-damage checkpoints and repair, epigenetic and transcriptional programs, microenvironmental cues, and circadian regulation. Yet, despite their diversity, these signals converge on a common bottleneck: They reshape cell-cycle progression [1,2]. For this reason, the cell cycle provides the fundamental axis linking intracellular regulation to population-level dynamics.

The cell cycle controls the basic rhythm of cell birth: Individual cells grow, replicate their DNA, and divide by following an ordered sequence of phases—G_1_, S, G_2_, M [3]. At the molecular level, progression through these phases is orchestrated by cyclin-dependent kinases (CDKs) and their cyclin partners [4,5], which are regulated by a complex network of molecular species that sets the tempo of phase transitions. Cyclins are synthesized and degraded in a phase-dependent manner, and by binding and activating CDKs, they generate pulses of kinase activity that trigger key events such as commitment to DNA replication and entry into mitosis [3].

When cell-cycle tasks are not properly completed—for instance, if DNA replication remains unfinished or DNA damage accumulates—checkpoint pathways engage and act on this same regulatory machinery [6,7], e.g., by inhibiting CDK activity or promoting cyclin degradation, thereby slowing or halting phase transitions and, when appropriate, diverting cells into reversible quiescence.

Viewed through this cell-cycle lens, deterministic models of cell population dynamics span a spectrum from phenomenological growth laws to progressively cell-cycle-structured and mechanism-informed formulations, reflecting a gradual resolution increase.

Over the past decades, a wide range of mathematical models have been developed to describe how the number and composition of cells in a population change as a consequence of cell-cycle dynamics. These models largely differ in how they represent population heterogeneity, how they specify the cell division process, and how they incorporate regulatory mechanisms and external perturbations.

Early modeling efforts relied on purely phenomenological approaches that aimed solely at reproducing the observed temporal evolution of whole cell populations [8]. These models typically fit simple mathematical curves to bulk growth data, without incorporating intracellular information. We therefore begin with these unstructured “black box” models—useful for fitting data, but largely silent on mechanism.

As experimental access to single-cell physiology and molecular regulation expanded, modeling has progressively moved toward more structured and cell-cycle-aware formulations.

**Fig. 1.**
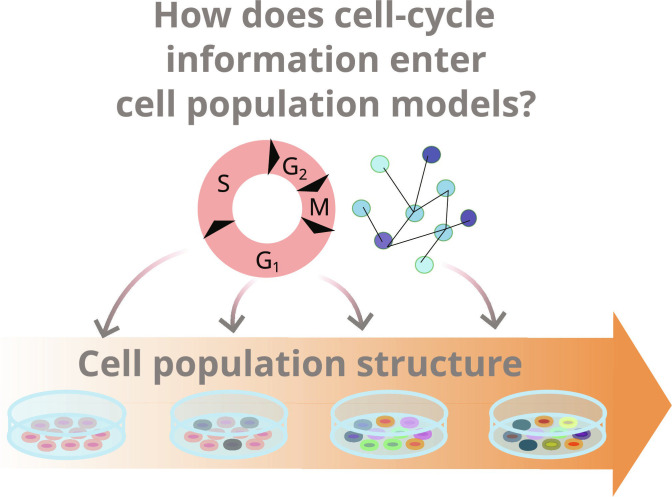
Guiding question of the review. This review asks how cell-cycle information can be translated into deterministic models of cell population dynamics across different population structures.

Accordingly, this review asks how information on cell-cycle progression can be used to inform deterministic models of cell population dynamics across different levels of population resolution (Fig. 1).

Across this path, modeling approaches mainly differ along 2 conceptual axes: (a) how deeply population resolution is represented, from unstructured descriptions of total cell numbers to formulations that resolve cell-cycle structure through discrete compartments or continuous state distributions, and (b) how deeply cell division is specified, from phenomenological effective rates to mechanistic couplings constrained by cell-cycle biochemical dynamics.

Given axis (a), we organize the review around 3 increasingly expressive ways of representing population heterogeneity: unstructured populations (Unstructured Models: Cycle-Agnostic Phenomenological Descriptions section), discretely structured (compartment-based) populations (Discretely Structured Models: From Cycling-Quiescent Partitions to Phase-Resolved Compartments section), and continuously structured (density-based) populations (Continuously Structured Models: Heterogeneity-Resolved Population Dynamics section). We will see that these model classes naturally map onto increasingly informative experimental measurements.

Within each class, we move along axis (b), from phenomenological effective terms to mechanistic couplings in which population dynamics are linked to cell-cycle regulation, as constrained by the chosen population structure. Along this axis, mechanistic cell-cycle descriptions may range from detailed network models, such as the classical Novak–Tyson frameworks, to reduced modular descriptions, such as the Gérard–Goldbeter models [9]. Although an in-depth review of intracellular cell-cycle network formulations is beyond our scope, we will refer back to these models when discussing how biochemical regulation can be folded into population-level descriptions.

Given our focus on cell-cycle-based information, we restrict our scope in 3 ways: First, the temporal dimension remains our preferential modeling axis, leaving a discussion of spatial effects beyond our scope. Second, we prioritize deterministic formulations, as they provide the structural backbone of most modeling frameworks, although we will briefly discuss their limitations and indicate where stochastic or hybrid methods are required. Third, we limit our analysis to population dynamics driven by cell division and loss, excluding long-term differentiation into distinct phenotypic lineages. This restriction still covers a broad class of systems, including cultured cell lines, tumor populations treated as a single proliferative lineage, and expanding immune or progenitor populations under conditions where stable lineage diversification is limited.

Eventually, we outline open challenges and suggest promising developments driven by increasingly rich single-cell datasets, recent advances in couplings between cell-cycle and population models, and computational methods.

## Unstructured Models: Cycle-Agnostic Phenomenological Descriptions

**Fig. 2.**
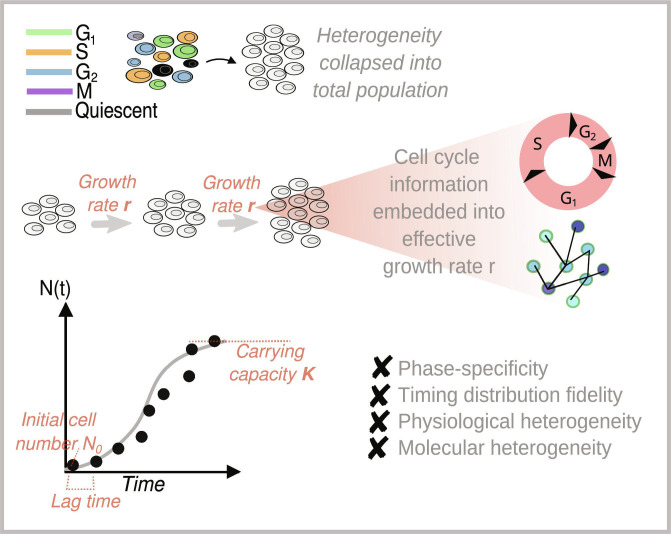
Unstructured cell population models. Cell-to-cell heterogeneity is aggregated into total population size, with dynamics described by an effective growth law (gray curve) parameterized by phenomenological coefficients (parameter examples highlighted in red). Cell-cycle information can enter unstructured models through an effective growth rate, as illustrated by the pink shaded inset. These models lack phase specificity, cannot generate faithful proliferation-timing distributions, and do not resolve physiological or molecular heterogeneity (bottom-right checklist).

Early quantitative descriptions of cell population growth were developed in settings where only coarse, whole-population observables were accessible. In many classical growth experiments, the available data consisted of total cell numbers, total biomass, or optical density, obtained from cell counters, spectrophotometers, or plate readers. Despite their early origins, such aggregate measurements remain widely used in high-throughput settings, including microbial growth characterization, industrial bioprocess monitoring, and large-scale drug screening assays [10,11]. In such contexts, no information is available on the internal state, physiology, or cycling status of cells. Consequently, modeling approaches focus on simple descriptions of bulk population expansion. These unstructured growth models are typically based on a low-dimensional population state—e.g., a single total cell number *N*(*t*)—and all cell-cycle mechanisms are lumped into one or a few effective parameters (Fig. 2).

The simplest and best-known unstructured growth law is given by [12,13]:

$$\frac{\mathrm{dN}}{\mathrm{dt}}=\mathrm{rN},$$ (1)

which yields an exponential population growth. Here, the effective growth rate *r* is typically estimated by fitting the model to bulk growth data such as the total cell number.

Experimental growth curves often deviate from such exponential expansion, because proliferation typically slows down as density increases and nutrients or space becomes limiting [14]. This motivated density-dependent model approaches such as the logistic one [15],

$$\frac{\mathrm{dN}}{\mathrm{dt}}=\mathrm{rN}-\frac{{\mathrm{rN}}^{2}}{K},$$ (2)

where *r* sets a net growth rate, while *K* acts as an effective population carrying capacity—summarizing crowding and environmental limitations. Although such forms can sometimes be derived from resource-limitation issues [16], in practice both *r* and *K* are treated as experiment-specific parameters inferred from bulk growth curves [17,18]. When nutrients are directly measurable, a complementary class of unstructured models replaces *K* with explicit dynamics for a limiting substrate concentration [13,19].

Since empirical growth curves often show asymmetric saturation or lag-like dynamics, Gompertz, Richards, and Baranyi–Roberts models are commonly used to capture these deviations from simple logistic behavior [20–22].

Cooperative growth at low density, known as the Allee effect, can be represented phenomenologically by an effective density threshold below which net growth is reduced or negative [23].

Importantly, in many settings, bulk population curves reflect not only cell production but also cell loss. The 2 contributions can be made explicit by decomposing the net rate *r* into distinct birth (*b*) and death (*d*) rates so that the simple exponential model becomes

$$\frac{\mathrm{dN}}{\mathrm{dt}}=\left(b-d\right)\;N.$$ (3)

Bulk growth readouts, such as longitudinal cell counts, optical density, confluency measurements, biomass, and colony-forming unit (CFU)-based curves, can be used to estimate effective parameters of unstructured growth laws, such as the net expansion rate r or the carrying capacity K, with doubling-time estimates providing a coarse summary of the same net kinetics [24,25]. However, in a birth–death decomposition, bulk trajectories alone constrain only their combination b−d, and separating the 2 contributions therefore requires adding viability/death readouts—such as live/dead staining or temporal assays of dead-cell accumulation [26,27]. A summary of experimental readouts associated with unstructured models, including birth–death formulations, is provided in Table 1.

**Table 1.** State variables represented in cell-cycle informed population models and common matching experimental observables and techniques. Representative references indicate methodological papers, protocols, or exemplary studies in which the corresponding experimental techniques are used to measure the listed observable.

Model state feature	Observable quantity	Experimental techniques
Total cell abundance	Bulk cell abundance	Cell counts; optical density; confluency imaging; biomass measurements; CFU growth assay [193,194]
Cell viability	Live/dead cell abundances	Viability assays; live/dead staining; temporal dead-cell accumulation assays; apoptosis assays [195]; clonogenic/CFU survival assays [196]
Cycling status	Cycling/proliferating cell abundances	Ki67 staining and BrdU immunostaining [197]; EdU/BrdU incorporation and label-retention assays [198];
Cell-cycle phase	Phase-specific cell abundances	DNA-content flow cytometry; DNA-content/BrdU or DNA-content/EdU flow cytometry; phase-specific markers; phospho-histone H3 staining [199]; FUCCI phase reporters; pulse–chase labeling with live-cell imaging [200]
Structuring cell mass variable	Population distribution of cell mass	Suspended microchannel resonator; quantitative phase imaging/microscopy; time-lapse quantitative microscopy; microfluidic single-cell measurements [147]
Structuring cell-size variable	Population distribution of cell size	Coulter counter; flow-cytometry forward scatter; image-based cell-size measurements; time-lapse quantitative microscopy; microfluidic or long-term live-cell imaging [119,145]
Structuring cell-age variable	Population distribution of cell age	Lineage-resolved time-lapse microscopy; long-term live-cell imaging with single-cell tracking [201,202]
Structuring molecular variable	Population distribution of molecular state/activity	Snapshot single-cell molecular measurements by quantitative immunofluorescence and flow cytometry [203]; RNA-FISH [204]; single-cell RNA sequencing [205]
Intracellular regulatory dynamics	Time-resolved molecular trajectories	Live-cell imaging with fluorescent molecular reporters and biosensors [206–208]

In some cases, it is nevertheless possible to assume negligible cell loss so that b≈r. For instance, during early expansion of a healthy culture, viability is high and d≪b, so omitting *d* may be reasonable. Predominantly cytostatic perturbations—such as serum starvation or CDK4/6 inhibition—can be represented mainly through reduced *b*, whereas cytotoxic treatments, irradiation, nutrient- or stress-induced loss of viability, or immune-cell contraction requires an explicit death term.

External perturbations typically enter these models phenomenologically by allowing rates (e.g., net growth, birth, and death) to depend on an external input *u*(*t*); for instance, $r=r\;\left(u\left(t\right)\right)$. *u(t*) may represent a controlled intervention (e.g., drug dosing) or an environmental variable that acts by modulating the aggregate proliferation and/or loss processes [28].

However, cell-cycle information can potentially enter effective rates such as *r*, as highlighted in Fig. 2, either in a data-driven or mechanistic way. Cell-kinetic readouts can provide independent estimates of proliferative timescales that help constrain or interpret effective rates beyond bulk-curve fitting alone. For instance, time-lapse lineage tracking, optionally combined with FUCCI reporters, estimates inter-division or phase-residence times; snapshot FUCCI imaging or DNA-content flow cytometry estimates cell-cycle phase composition; Ki67 staining estimates the cycling fraction of the population; and 5-ethynyl-2′-deoxyuridine (EdU)/5-bromo-2′-deoxyuridine (BrdU) incorporation estimates S-phase entry or occupancy.

As an example, Begg et al. [29] introduced a BrdU/DNA-content flow-cytometry method to estimate S-phase duration and potential doubling time $T_{\mathrm{pot}}$ from a single sample; related tumor studies used BrdU labeling with DNA-content flow cytometry to assess proliferative kinetics in human tumors [30]. Eidukevicius et al. [31] further used BrdU flow cytometry to estimate cell-cycle time and growth fraction from tumor samples, translating snapshot clinical biopsies into dynamic proliferative descriptors relevant to tumor aggressiveness.

A stronger form of coupling consists of linking bulk growth to cell-cycle mechanism by letting a molecular state variable or regulatory network inform an effective rate. In this way, experimental growth curves can be interpreted through the lens of upstream regulatory dynamics, and the same regulatory layer can be used to predict untested perturbation conditions.

Alarcón et al. [32] provide a clear example of such an upscaling strategy, in which a logistic population model is coupled to a dynamic CDK ordinary differential equation (ODE) network derived from [33]. When a network-generated cell-cycle variable reaches a threshold, cell division occurs—exploiting an event-gated strategy as in Fig. 6B. This defines an effective cell-cycle period $T_{\mathrm{cyc}}\left(t\right)$ that can be mapped onto the net proliferation rate via

$$r\left(t\right)\approx \frac{\text{ln}2}{T_{\mathrm{cyc}}\left(t\right)}.$$ (4)

In this way, the bulk growth rate can be interpreted in terms of CDK-regulatory dynamics that set the effective cell-cycle duration.

One can also let the proliferation term depend continuously on intracellular regulatory variables *z*(*t*), i.e., $r=r\left(z\left(t\right)\right)$ (as in Fig. 6C). For example, He et al. [34] coupled estrogen receptor (ER)/cyclin-dependent kinase 4 and 6 (CDK4/6)/retinoblastoma protein (Rb)–E2F transcription factor (E2F) network dynamics to MCF-7 breast cancer population growth, modulating proliferation under endocrine and CDK4/6-inhibitor treatments. The regulatory module was used for in silico prediction of drug combinations, thereby guiding experimental design.

However, network closures are rarely paired with unstructured models, because the absence of structure makes different regulatory scenarios collapse onto one or a few effective rates and thus distinct mechanisms may yield indistinguishable growth curves.

In sum, unstructured population models recapitulate the main macroscopic features of bulk population growth curves but do not resolve cell-cycle progression and thus cannot resolve phase-specific effects (see checklist in Fig. 2). Because the population is represented only by an aggregate variable, these models do not track cells from birth to division and therefore cannot define division-time distributions. Their low population state also prevents them from resolving cell-to-cell heterogeneity unless additional structure is introduced. This motivates explicit representation of cell-cycle state at the population level, leading to the structured formulations reviewed next.

## Discretely Structured Models: From Cycling-Quiescent Partitions to Phase-Resolved Compartments

A natural step beyond unstructured growth laws is to make cell-cycle progression an explicit population state variable by resolving cells into a finite number of compartments and tracking the corresponding subpopulation sizes through balance equations. The resulting discrete compartment models are attractive because they remain low-dimensional systems while enabling phase-level predictions—e.g., time-dependent phase composition, synchrony, and phase-specific responses.

In this section, we present a progression from the simplest discrete partitions to more cell-cycle-informed formulations; for each approach, we briefly indicate the experimental readouts that most directly constrain the corresponding models, as well as how cell-cycle information can enter the same models, along with typical experimental applications.

### Cycling-quiescent compartment models

The simplest discretely structured description distinguishes only between cycling—i.e., proliferative—and noncycling—i.e., quiescent/arrested—cells, optionally with a dead compartment. Letting $P=\left\{P,Q\right\}$ denote the set of compartments, one can call $N_{P}\left(t\right)$ and $N_{Q}\left(t\right)$ respectively the cycling and noncycling subpopulation sizes so that the total population size is

$$N\left(t\right)=\sum_{p\in P}N_{p}\left(t\right)=N_{P}\left(t\right)+N_{Q}\left(t\right).$$ (5)

A minimal model then reads

$$\frac{{\mathrm{dN}}_{P}}{\mathrm{dt}}=\left(\beta -k_{P\to Q}-d_{P}\right)N_{P}+k_{Q\to P}\;N_{Q},$$ (6)

$$\frac{{\mathrm{dN}}_{Q}}{\mathrm{dt}}=k_{P\to Q}\;N_{P}-\left(k_{Q\to P}+d_{Q}\right)N_{Q},$$ (7)

where *β* is an effective division rate within the cycling pool, $k_{P\to Q}$ and $k_{Q\to P}$ are exchange rates between cycling and noncycling states, and $d_{P},d_{Q}$ are effective compartment-specific death rates.

Models of this type are widely used in contexts where the dominant question is whether growth inhibition is due to (a) reduced cycle throughput within the cycling pool (β), (b) diversion into noncycling states ($k_{P\to Q}$), or (c) loss of viability through cell death ($d_{P},d_{Q}$), which is particularly relevant in oncology where treatments may induce cell-cycle arrest and thus enrich for slow-cycling subpopulations [35,36]. Furthermore, quiescence emerges in diverse contexts as it regulates long-term tissue maintenance in stem cell homeostasis as well as microbial dormancy and persistence under stress, and bioprocessing systems where environmental limitations can induce growth arrest. Accordingly, proliferating–quiescent decompositions are widely used not only in tumor-growth modeling but also in stem cell systems [37], microbial persistence models [38], and bioprocessing applications [39].

Classical 2-compartment tumor models include the Gyllenberg–Webb framework and subsequent developments that explicitly connected them to Gompertzian macroscopic growth behavior [40–42]. In treatment-oriented formulations, one often introduces an additional living compartment, to represent a pharmacologically “blocked” or “recruited” subpopulation, so that cytostatic and recruitment effects are captured explicitly rather than being absorbed into effective exchange rates; this yields closely related 3-compartment control models [43,44]. In this framework, external perturbations can be represented phenomenologically by allowing selected rates to depend on an external input *u*, which may represent either a controlled intervention (e.g., drug dosing or growth-factor administration) or an environmental variable. In practice, *u* does not act directly on cell numbers, but modulates specific fluxes between compartments. For instance, pharmacological action can be incorporated by letting $\beta =\beta \left(u\right)$ to capture cytostatic effects that reduce division within the cycling pool, $d_{P}=d_{P}\left(u\right)$ to model cytotoxicity that increases death among proliferating cells, or $k_{P\to Q}=k_{P\to Q}\left(u\right)$ to describe drug-induced arrest and diversion into a noncycling state. The functional dependence on *u* is typically chosen phenomenologically—e.g., linear, saturating, or sigmoidal forms—and calibrated from longitudinal bulk measurements and/or state-specific marker readouts when available, or specified from prior knowledge in control-oriented studies [43–46].

Note that purely bulk experimental $N\left(t\right)=N_{P}+N_{Q}$ time series often constrain only a net growth combination and cannot uniquely separate $\beta ,k_{P\to Q},k_{Q\to P}$, whereas even coarse proliferation markers or cycling-fraction readouts can help identify fluxes between $N_{P}$ and $N_{Q}$. For example, Tyson et al. [47] estimated division, quiescence-entry, and death rates in a 2-compartment model by combining longitudinal cell counts with time-lapse imaging that constrains the fraction of cells entering and leaving quiescence, thereby disentangling proliferative fluxes from net population growth.

In general, these 2-state partitions align most naturally with minimal readouts that report the cycling fraction rather than full phase composition, such as Ki67-based immunostaining (cycling versus noncycling), short EdU/BrdU pulse incorporation to quantify the fraction of cells entering S phase over a fixed window, and time-lapse measurements of division events (mitotic index / division frequency). A list of experimental readouts that typically constrain quiescent-proliferative partitions is reported in Table 1. Cycling–quiescence exchange kinetics, i.e., $k_{P\to Q}$ and $k_{Q\to P}$, have been quantified within such compartment models by fitting Ki67-positive/negative compositions and their time evolution [48,49]; when combined with longitudinal total cell counts, the same framework can be calibrated in absolute terms by additionally constraining net population growth [50]. In a related spirit, Glauche et al. [51] used a simple quiescent–activated switching model to interpret pulse–chase proliferation labeling data (e.g., BrdU incorporation time courses) and constrain activation and return-to-quiescence fluxes. Imaging-derived estimates of the proliferative status (P:Q ratio), e.g., via BrdU-calibrated positron emission tomography (PET) biomarkers, have been used to constrain Q/P balances and their modulation under therapy [52].

In turn, bulk viability assays or apoptosis markers (live/dead, clonogenic/CFU-type readouts) can inform $d_{P},d_{Q}$, enabling inference of compartment-specific division and death contributions that are otherwise confounded in Q/P readouts [53].

For a mechanistically grounded description, transition rates can be coupled to cell-cycle network models that generate commitment to proliferation and exit timings at the single-cell level. To incorporate a Q/P transition mechanism, it is in principle sufficient to use cell-cycle commitment switches that act upstream of the full CDK oscillator, such as the Rb–EF module that determines entry into a proliferative regime before progression through the subsequent cell-cycle phases occurs. Yao et al. [54] demonstrated experimentally that the Rb–E2F module operates as a bistable switch at the restriction point, converting graded mitogenic inputs into an all-or-none commitment to proliferation. Bulding on this, Pandey and Vinod [55] developed a mechanistic CDK/Rb–E2F-based bistable switch that captures the reversible transition between quiescence and proliferation and generates commitment timings that could be used to inform effective transition terms at the population level. Relying on such bistable commitment modules, one practical route is to define transition propensities by coarse-graining threshold/hysteresis events—e.g., start/restriction-point crossing or exit from the proliferative attractor—and translating them into effective Q↔P fluxes (Fig. 6B).

Lee et al. [56] further showed that activation dynamics of the Myc–Rb–E2F module generate distributions of cell-cycle commitment times that can be recast into effective Q→P entry descriptors (e.g., a transition rate and a time delay). Note that such a mapping compresses the intracellular dynamics into static effective flux terms (as in Fig. 6A), rather than letting it modulate those terms in real time.

Under an external input (e.g., growth factors, stress, or drugs) acting on the mechanistic switch, changes in the output of the regulatory module (e.g., shifts in threshold-crossing times) can be mapped onto corresponding modulations of the effective Q→P and P→Q fluxes.

However, multiscale couplings, in which a commitment variable or network is embedded in-line so that Q↔P switching times emerge directly as population-level fluxes, remain rare. Mechanistic closures are most often exploited in descriptions that retain some explicit representation of cell-cycle position: The Q/P partition still treats the entire cycling compartment as dynamically homogeneous, i.e., it assumes that cells within *P* are well mixed with respect to their position in the cell cycle and that perturbations act uniformly across the compartment.

In reality, most regulatory and pharmacological effects operate as localized gates at specific cell-cycle transitions—for instance, at the restriction point in late $G_{1}$, during DNA replication in S, or at the spindle-assembly checkpoint in M [57–59]—and are therefore difficult to represent within a single pooled *P* state.

Overall, Q/P partitions predict population dynamics by balancing cycling and noncycling states. They aid experimental design by showing how pairing longitudinal counts with cycling or viability readouts allows one to infer unobservable transition rates. This makes it possible to distinguish reversible dormancy from irreversible cell loss, although these models cannot resolve phase-specific effects.

This limitation motivates discretely structured formulations that resolve the cycling population into a small number of cell-cycle-related states.

### Phase-compartment models

Many discretely structured approaches resolve the cycling pool into a small number of cell-cycle-related compartments, ranging from more coarse splits (e.g., G_1_ versus S/G_1_/M) up to the canonical phases G_1_, S, G_2_, and M, yielding minimal phase-resolved descriptions. These formulations are useful when the scientific question concerns phase-specific effects, and when available data resolve coarse cell-cycle classes. In this setting, let compartments be indexed by p∈P, with $N_{p}\left(t\right)$ denoting the number of cells in compartment *p* and $N\left(t\right)=\sum_{p\in P}N_{p}\left(t\right)$ the total population size. In a classical formulation, the phase-compartment balance equations can be written as

$$\frac{{\mathrm{dN}}_{p}}{\mathrm{dt}}=\sum_{q\in P}\nu_{q\to p}\;k_{q\to p}\;N_{q}-\sum_{r\in P}k_{p\to r}\;N_{p}\;-\;\delta_{p}\;N_{p},$$ (8)

where $\nu_{q\to p}$ encodes stoichiometry (e.g., 2 $G_{1}$ cells produced per mitosis), $k_{q\to p}$ are effective phase-transition propensities, and $\delta_{p}\geq 0$ represents an effective loss rate from compartment *p*; if required, one may also track an explicit dead compartment *D*(*t*) with $\frac{\mathrm{dD}}{\mathrm{dt}}=\sum_{p\in P}\delta_{p}N_{p}-\gamma D$ while keeping the same phase-transition backbone. For the canonical 4-phase split $P=\left\{G1,S,G2,M\right\}$, the model above takes the explicit form

$$\frac{{\mathrm{dN}}_{G1}}{\mathrm{dt}}=2\;k_{M\to G1}\;N_{M}-k_{G1\to S}\;N_{G1},$$ (9)

$$\frac{{\mathrm{dN}}_{S}}{\mathrm{dt}}=k_{G1\to S}\;N_{G1}-k_{S\to G2}\;N_{S},$$ (10)

$$\frac{{\mathrm{dN}}_{G2}}{\mathrm{dt}}=k_{S\to G2}\;N_{S}-k_{G2\to M}\;N_{G2},$$ (11)

$$\frac{{\mathrm{dN}}_{M}}{\mathrm{dt}}=k_{G2\to M}\;N_{G2}-k_{M\to G1}\;N_{M}.$$ (12)

Note that, although not represented here for simplicity, noncycling behavior is often described either by an explicit quiescent compartment connected to $G_{1}$ with entry/exit kinetics or by modulating the $G_{1}\to S$ transition to represent checkpoint-controlled gating [60–62].

When studies involve controlled perturbations such as drug dosing protocols, phase-transition rates are naturally allowed to depend on treatment—i.e., $k_{q\to p}\left(u\left(t\right)\right)$ carries a dependence on an external input *u(t*)—and, when needed, one introduces phase-specific death terms that depend as well on *u*(*t*) to describe cytotoxic effects. In this way, treatment-driven phase redistribution can be predicted and fitted [45,46,63–65]. Phase-compartment models have been extensively studied within optimal control frameworks, where therapies modulate selected transition or killing terms and dosing protocols are optimized to maximize tumor reduction subject to toxicity constraints [45,46,63,64].

Since distinct perturbations (e.g., drug actions) can generate similar total-growth curves *N*(*t*), and total population counts only constrain $\sum_{p}N_{p}\left(t\right)$, separating mechanisms typically requires at least partial phase-resolved readouts (see Table 1) that constrain phase-specific abundances and thereby help infer the relative magnitudes of the $k_{q\to p}$ rates. Note also that bulk doubling times reflect the overall cycle throughput and can therefore constrain only an aggregate timescale set by the combined $k_{q\to p}$ rates.

Transition rates are thus often treated as condition-specific constants and estimated from phase-resolved experimental readouts: DNA-content flow-cytometry time-courses resolve $G_{1}$, S, and $G_{2}/M$ fractions over time [66]. When combined with total cell counts and interpreted under a specified compartmental model, these data can be used to reconstruct phase-abundance trajectories $N_{p}\left(t\right)$ and constrain effective transition rates $k_{q\to p}$ [44,63]. In parallel, EdU/BrdU pulse incorporation reports the fraction of cells undergoing DNA synthesis during the labeling window, thereby constraining the $G_{1}\to S$ transfer term $k_{G1\to S}N_{G1}$. Pairing DNA-content and pulse-labeling readouts enables bivariate localization of labeled cells along the $G_{1}$–S–$G_{2}/M$ axis, improving estimates of phase transit times and effective transfer rates [67,68]. Time-lapse FUCCI trajectories directly report single-cell phase-residence times and transition events in live cells. In compartmental models, these measurements can be compressed into effective phase-transition rates by using the inverse of the observed average residence times, for example, $k_{G1\to S}\approx 1/\langle T_{G1}\rangle$. In FUCCI implementations that resolve $G_{1}$ but not S, $G_{2}$, and M as separate phases, the post-$G_{1}$ portion of the cycle can instead be treated as a combined $S/G_{2}/M$ block, yielding an effective exit rate $k_{S/G2/M\to G1}\approx 1/\langle T_{S/G2/M}\rangle$ [69,70].

As mentioned for the quiescent-proliferative framework, fixed-cell Ki67 immunostaining primarily constrains the cycling fraction, and therefore can complement DNA-content/FUCCI readouts by disentangling quiescence from G_1_ phase cells. Finally, phospho-histone H3 staining provides an endpoint measure of mitotic enrichment, i.e., a direct constraint on the M-phase occupancy—commonly referred to as mitotic index. Combined with an independent estimate of M-phase duration or overall throughput, this can be used to infer mitotic exit and disentangle mitotic entry from exit (e.g., informing $k_{M\to G1}$ versus $k_{G2\to M}$) in phase-compartment models [71,72].

Lonati et al. [73] provide an exemplary case study showing how phase-resolved experimental readouts can calibrate a compartmental model of radiation-induced cell-cycle perturbation. Flow-cytometry measurements after irradiation were used to estimate dose-specific changes in transition rates, including the $G_{2}\to M$ transition, reproducing time-dependent cell redistribution. In this way, they highlighted time windows in which surviving cells are expected to accumulate in radiosensitive phases, suggesting a temporal optimization of radiotherapy schedules.

**Fig. 3.**
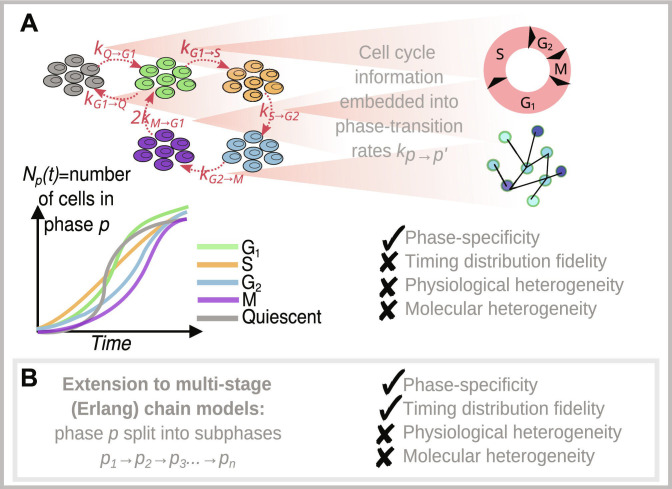
Discretely structured population models. (A) Phase-compartment models. Cells are represented as compartments, with different colors indicating their cell-cycle status: G_1_ (green), S (orange), G_2_ (blue), M (purple), and quiescent (gray). The plot shows an example of bulk phase-resolved population growth curves. Effective transition rates between compartments are highlighted in red. Cell-cycle information can enter discretely structured population models through these transition rates, as illustrated by the pink shaded inset. These models resolve phase specificity, but cannot generate faithful phase-timing distributions, and do not resolve physiological or molecular heterogeneity (bottom-right checklist). (B) Multi-stage models. Phase-compartments are split into multiple sub-phases. These models are capable of generating faithful phase-timing distributions (bottom-right checklist).

At this level, the cell cycle still enters the model only implicitly through condition-specific effective parameters. As a minimal enrichment with mechanistic information, one can couple rates $k_{q\to p}$ with cell-cyle dynamics (as highlighted in Fig. 3). This coupling can rely, for instance, on a coarse cell-cycle regulatory marker (e.g., cyclin levels or checkpoint status) so that phase transitions are gated by a continuous molecular state (as in Fig. 6C). A number of studies sit close to this regime, without propagating a full cyclin–CDK dynamic network. For instance, a molecule-gated form of this continuous-modulation strategy was used by Eladdadi and Isaacson [74], who developed a 3-compartment cell-cycle model in which the $G_{1}\to S$ and $S\to G_{2}/M$ transition rates depend on epidermal growth factor receptor (EGFR)/human epidermal growth factor receptor 2 (HER2) receptor-complex signaling through saturating functions. Their framework enabled in silico drug screening to select targeted combination treatments to be experimentally tested.

Similarly, but with the gating variable generated by a dynamic intracellular network, Venkatasubramanian et al. [61] implemented continuous modulation of transition rates by making $k_{G_{1}\to S}$ depend on an energetic/metabolic state, such as adenosine 5′-triphosphate (ATP), thereby constraining $G_{1}\to S$ progression. An explicit dynamical network module has also been introduced in a pharmacokinetic/pharmacodynamic (PK/PD) cell-cycle model, which relies on a drug-induced damage variable to gate progression/division events via threshold-type rules [75], as in Fig. 6B.

In principle, one may let transition propensities depend on a full mechanistic cyclin–CDK model, writing $k_{q\to p}=k_{q\to p}\left(z\left(t\right)\right)$, with *z*(*t*) evolving according to a dynamical system:

$$\frac{\mathrm{dz}\left(t\right)}{\mathrm{dt}}=C\left(z\left(t\right)\right),$$ (13)

where *C* denotes the regulatory dynamics. In this framework, perturbations can enter upstream at the regulatory level by letting *C* depend also on an external input *u*, i.e., $C\left(z\left(t\right),u\left(t\right)\right)$: They propagate through *C* and thereby reshape the regulatory trajectory *z*(*t*), which in turn modulates transition rates through $k_{q\to p}\left(z\left(t\right)\right)$. Mechanistic modules that describe checkpoint-specific mechanisms—for instance, spindle-assembly checkpoint models [76]—are therefore naturally suited to inform these formulations.

In conclusion, discrete phase-compartment formulations expose enough population structure to represent phase-specific effects. However, because each compartment is internally homogeneous, cell-cycle mechanisms are coarse-grained into transition propensities acting identically on all cells in the same compartment. When these propensities are reduced to constant mean transition rates—as in Fig. 6A—the model fixes only the mean phase-residence time and implies an exponential residence-time distribution with coefficient of variation equal to one [77]. This limits timing-distribution fidelity, since experimentally measured phase durations are typically narrower and more structured [77–79].

In-line intracellular modules can make transition propensities time-dependent and improve the regulatory interpretation of phase transitions. However, if this modulation is shared by the whole compartment, cells with different residence ages remain indistinguishable. Thus, purely compartmental ODE couplings do not by themselves recover faithful phase-residence time distributions.

Figure 3A summarizes discrete phase-compartment models, highlighting that they can represent phase-specific effects at the compartment level, but cannot recapitulate faithful timing distributions or either physiological or molecular cell-to-cell variability.

Multi-stage Erlang models, reviewed next, address the specific timing limitation of phase-compartment models by replacing a single memoryless phase step with a sequence of substages that approximates more realistic residence-time distributions.

### Multi-stage (Erlang) models

A minimal way to address the artifactual phase-transition timings produced by memoryless phase compartments, while retaining a finite-dimensional Markovian ODE description, is a phase-type (Erlang) representation (Fig. 3B). Here, a phase p∈P is replaced by a short chain of *k* serial substates $p^{\left(1\right)},\ldots ,p^{\left(k\right)}$ with identical exponential transitions [77–82]: Cells progress sequentially through these substates before exiting the phase so that the total phase-residence time becomes the sum of *k* exponential waiting times. For simplicity, we illustrate the construction—which holds for any phase—by splitting G_1_ only: One replaces the single compartment G1 by the chain $G1^{\left(1\right)},\ldots ,G1^{\left(k\right)}$, with $N_{G1^{\left(i\right)}}\left(t\right)$ denoting the number of cells in substate $G1^{\left(i\right)}$. The resulting balance equations, which in this specific case recapitulate the G_1_–S transition, read

$$\frac{{\mathrm{dN}}_{G1^{\left(1\right)}}}{\mathrm{dt}}=2\;k_{M\to G1}\;N_{M}-k_{G1}\;N_{G1^{\left(1\right)}},$$ (14)

$$\frac{{\mathrm{dN}}_{G1^{\left(i\right)}}}{\mathrm{dt}}=k_{G1}\;N_{G1^{\left(i-1\right)}}-k_{G1}\;N_{G1^{\left(i\right)}},\;i=2,\ldots ,k,$$ (15)

$$\frac{{\mathrm{dN}}_{S}}{\mathrm{dt}}=k_{G1\to S}\;N_{G1^{\left(k\right)}}-k_{S\to G2}\;N_{S}.$$ (16)

Here $k_{G1}$ denotes the common progression rate between successive G_1_ subcompartments, whereas $k_{G1\to S}$ represents the transition rate from the final G_1_ substate $G1^{\left(k\right)}$ to the S phase. The remaining phase equations (for $N_{G2}$ and $N_{M}$) are unchanged from the 4-phase system. This yields an Erlang-distributed dwell time with the same mean but variance reduced by a factor *k* and thus captures more realistic phase timing variability—improving fits and dynamical behavior while still retaining an ODE structure [77,78,82,83]. From a data-driven viewpoint, these extensions become particularly justified when phase-duration statistics—e.g., from time-lapse single-cell tracking or phase-resolved reporters—are available and show clear deviations from exponential waiting times [78]; without such information, increasing *k* can quickly introduce non-identifiability.

In drug-perturbation settings, linear-chain phase-compartment models have implemented treatment action as dose-dependent phenomenological modulation of substate progression rates, i.e., $k_{p}=k_{p}\left(u\left(t\right)\right)$, with *u*(*t*) representing an external modulator, allowing fits to time-resolved phase fraction and growth data [84]. More broadly, utilizing multi-stage transit chains is a well-established paradigm in mathematical pharmacology to introduce a structural time-lag in a population’s response to therapy [85,86]. Indeed, in the context of cell-cycle modeling, this Erlang framework has been successfully applied to capture the gradual onset and recovery kinetics observed in experimental phase fractions [84], overcoming the artificial, instantaneous responses inherent to standard phase-compartment formulations.

Besides their capability to capture experimental dwell-time distributions, mechanistic coupling of these models to molecular networks is uncommon: The multi-stage chain is a surrogate that does not represent biochemical or checkpoint states. Thus, multi-stage models should be viewed mainly as a phenomenological refinement of phase-transition timing. They introduce an apparent residence-time memory, but they still do not resolve continuous physiological or molecular heterogeneity—as reported in the checklist of Fig. 3B.

In sum, discretely structured compartment models occupy an intermediate point between unstructured growth laws and fully continuous physiologically structured formulations, making cell-cycle position an explicit state variable. Even if linear-chain extensions improve phase timing-distribution fidelity, the homogeneous compartment representation still treats all cells within each substage as identical, thereby coarse-graining intra-phase heterogeneity. This motivates continuously structured population models, in which cells are explicitly distributed along a physiological coordinate associated with progression, such as age-in-phase, size, or molecular content.

## Continuously Structured Models: Heterogeneity-Resolved Population Dynamics

While discretely structured models are often sufficient to recapitulate bulk phase-resolved data, they collapse all within-phase progression into a single state and thus average over the continuously evolving physiological trajectories followed by individual cells. As a result, the dependence of population dynamics on cell heterogeneity is effectively lost. This becomes increasingly limiting in contexts where heterogeneity plays a key role—for instance, in drug-response studies, where age distributions can dictate population responses to targeted therapeutics [87], or in microbial growth studies, where single-cell growth and division-time variability has been shown to affect the resulting population growth rate [88].

Moreover, distinct sources of heterogeneity—in growth, progression, and fate—may produce indistinguishable compartment occupancies and total counts, thereby confounding mechanistic interpretation and limiting parameter identifiability.

Accordingly, experiments now provide distributional single-cell readouts–e.g., time-resolved size/age distributions and lineage-resolved trajectories—that simple compartment models cannot naturally handle. These data show that cells within the same population exhibit substantial heterogeneity in measurable physiological features—including size, mass, age, and cell-cycle progression—and that these quantities evolve continuously over time [89,90]. Concomitantly, time-lapse microscopy and flow-cytometry studies have demonstrated that division timing and probability are strongly modulated by the physiological state of a cell [89–92]. As a consequence, population-level growth and size homeostasis emerge from the aggregate of many individual trajectories through physiological state space, rather than from a single homogeneous growth process shared by all cells [93–95]. Structured population models aim to capture this complexity explicitly by representing the population as a distribution over one or multiple chosen physiological variables. In this framework, population dynamics is governed by 2 coupled processes: the progression of individual cells through physiological state space, and the occurrence of division—whose probability depends on such state. In its simplest form, this is achieved by formulating the problem in terms of partial differential equations that describe the time evolution of cell population density over a chosen physiological coordinate—e.g., mass, size, or age—and by coupling the evolution of such density to division events depending on the same physiological state [94–97]. Importantly, although the formulation is informed by physiological cell-to-cell heterogeneity, it does not track individual cell trajectories: It deterministically propagates the cell population density over the physiological state space. This provides a minimal framework for linking single-cell heterogeneity to population dynamics while retaining a deterministic formulation.

We begin this section with the basic structured population-balance formulation, encompassing size- and age-based variants, and then introduce cell-cycle resolution through phase-resolved compartmentalization.

### Structured population-balance models

The basic one-dimensional formulation of structured population-balance models [94,96], neglecting cell death, can be written in general form as a population-balance equation (PBE):

$$\begin{array}{c} \begin{array}{c} \frac{\partial F\left(x,\;t\right)}{\partial t}=\\ -\frac{\partial }{\partial x}\left[r\left(x\right)F\left(x,t\right)\right]-\gamma \left(x\right)\;F\left(x,t\right)+\int p\left(x|x^{\prime }\right)\;\gamma \left(x^{\prime }\right)\;F\left(x^{\prime },\;t\right)\;dx^{\prime }, \end{array} \end{array}$$ (17)

where $F\left(x,\;t\right)$ denotes the density of cells with physiological state *x* (e.g., mass/size/age) at time *t*.

The left-hand term, i.e., the time derivative, referred to as the transient term, captures the temporal evolution of $F\left(x,\;t\right)$ and thus vanishes at steady state, when the density reaches stationarity. The first right-hand term is the advection term, describing the transport of the population along the *x* axis—e.g., mass, volume, and age. Experimentally, this term reflects the physiological progression of individual cells, which move through size/mass/age space according to their individual growth trajectories. Here, *r*(*x*) describes, for instance, a rate of growth in size or age, i.e., $r\left(x\right)=\mathrm{dx}/\mathrm{dt}$: If cells increase their mass *x* at a rate *r*(*x*), the population density is correspondingly shifted toward higher *x* values; note that, in case *x* represents age, *r*(*x*) is typically equal to one, as cell age increases at unit rate in time. The last 2 terms on the right-hand side describe a sink and a source, which respectively remove and add cells to the population as a consequence of cell division. Here, $\gamma \left(x\right)$ describes the state-dependent division propensity, i.e., a division rate dependent, for instance, on size/mass/age *x*. Eventually, the partitioning kernel $p\left(x|x^{\prime }\right)$ describes how the state of a mother cell $x^{\prime }$ is mapped to the states of daughter cells at division: Cells are removed and reintroduced according to *p*, which describes where in state space newly born cells re-enter the population—e.g., at which size/mass/age they are generated. Depending on the nature of the structuring variable *x*, $p\left(x|x^{\prime }\right)$ takes different forms. If *x* represents a quantity partitioned at division—such as size or mass—this kernel describes the distribution of daughter states *x* produced by a mother in state $x^{\prime }$. Under mass/size conservation, it can be interpreted as the probability density that a mother of size/mass $x^{\prime }$ produces a daughter with size/mass *x* (with the complementary daughter receiving $x^{\prime }-x$) so that division depletes cells at larger sizes and “re-injects” newborn cells across a range of smaller size/mass values [93,94]. If instead *x* is reset at birth—most notably when *x* denotes age—then all daughter cells are created at a single reset state, typically *x* = 0. In this case, renewal does not generate a distributed source over x>0, but an influx through the boundary. As first demonstrated in [98], in such an age-structured formulation, birth renewal is imposed through the boundary condition

$$F\left(0,\;t\right)=2\int_{0}^{\infty }\gamma \left(a\right)\;F\left(a,\;t\right)\;\mathrm{da}.$$ (18)

rather than as a volumetric birth term.

The number of cells lying within any physiological interval $\left[x_{1},\;x_{2}\right]$ at time *t*—e.g., cells falling within a given size, mass, or age range—is recovered by integrating the density $F\left(x,t\right)$ over that interval,

$$\int_{x_{1}}^{x_{2}}F\left(x,\;t\right)\;\mathrm{dx}.$$ (19)

In particular, the total number of cells at time *t* is obtained by integrating over the entire admissible domain of the structuring variable, i.e., by setting $x_{1}=x_{\min}$ and $x_{2}=x_{\max}$, where $\left[x_{\min},\;x_{\max}\right]$ defines the full range of *x*.

The functional specification of the terms in Eq. (18) can vary substantially, because cells differ in how they grow, commit to division, and partition their contents. Hereafter, we thus describe how quantitative measurements can be used to specify the form of the 3 fundamental components of the PBE: *r*(*x*), $\gamma \left(x\right)$, and $p\left(x,\;x^{\prime }\right)$. For concreteness, we hereby consider the case where the structuring variable *x* represents cell size or mass, which provides a natural physiological coordinate for growth-division models. Age-based formulations and multi-structured variants can be recovered as particular cases of the same general framework.

Quantitatively, the growth law *r*(*x*) can be directly inferred from single-cell time-lapse quantitative microscopy, including measurements performed in microfluidic devices or long-term live-cell imaging setups, which provide longitudinal measurements of volume or mass for individual cells across generations [89,90,99]. These readouts allow estimation of both the mean growth law and its cell-to-cell variability under specific conditions [100,101]. The corresponding experimental techniques for measuring volume or mass as structuring variables are reported in Table 1.

Linear growth in cell volume has been used as a phenomenological approximation in some models of cell-cycle progression and size control [102,103]. However, a large body of single-cell experiments has shown that cell volume increases approximately exponentially over most of the cell cycle in a wide range of organisms. This behavior has been documented in bacteria [89–91,104], in budding yeast [105–108], and in mammalian cells [99,109]. Accordingly, the temporal evolution of cell volume is often modeled using an exponential law [110–113]:

$$V\left(t_{d}\right)=V_{0}\text{exp}\;\left(\frac{\text{ln}2}{T}t_{d}\right)$$ (20)

where $V_{0}$ is the volume at birth, $t_{d}$ is the time since the last division, and *T* is the volume-doubling interval—which can be estimated in a species- or condition-specific manner. Note if single-cell volume increases at a rate proportional to cellular biomass [114], and biomass concentration remains approximately constant, then both cell mass and volume grow exponentially [92,114]. Thus, if one adopts cell volume as the structural variable *x* in the PBE framework, the single-cell growth law *r*(*x*) will denote the rate of volume change for a cell of size *V* and will stem by deriving Eq. (20):

$$r\left(V\right)=\frac{\mathrm{dV}}{\mathrm{dt}}=\frac{\text{ln}2}{T}\;V$$ (21)

which will directly shape the advection term in Eq. (18). Note that, as shown in [111,112], the temporal volume form given by Eq. (20) implies a constant dilution rate for any intracellular protein,

$$d_{\text{dilution}}=\frac{1}{V}\frac{\mathrm{dV}}{\mathrm{dt}}=\frac{d}{\mathrm{dt}}\text{ln}V\left(t\right)=\frac{\text{ln}2}{T},$$ (22)

so that, in multiscale formulations where intracellular concentrations are modeled alongside population dynamics, an exponential *V*(*t*) also allows protein dilution to be represented consistently [100,101].

However, experiments have reported systematic deviations from strict exponential behavior at the single-cell level, for instance, in bacteria under nutrient limitation—whose growth rates shrink in a cell size-dependent manner, or in proliferating cancer cells under stress conditions such as metabolic inhibition or cytostatic drug action [99,100,112,115,116]. These scenarios motivate *r* forms that capture size-dependent slowing of growth, such as sublinear polynomial or saturating functions:

$$r\left(V\right)=\alpha V^{\beta }\;\text{with}\;\beta <1$$ (23)

or

$$r\left(V\right)=\alpha V\left(1-\frac{V}{V_{\max}}\right)$$ (24)

The role of resource availability in shaping single cell growth rates can be further embedded in population-balance models by including substrate- or nutrient-dependent growth laws [97,117]. Moreover, single-cell measurements have revealed fluctuations in growth rates even among cells of similar size, indicating a noisy relationship between growth and physiological state [100,101]. Stochastic extensions have been proposed in which the mass-dependent growth rate *r*(*x*) is allowed to fluctuate around a mean value [97,118].

The functional form of *r*(*x*) directly impacts the dynamics of the population: Experiments show that while strictly exponential growth maintains size heterogeneity, deviations toward linear or sublinear growth—often triggered by nutrient restriction—actively reduce population variability [108,119]. This is crucial in bioprocess applications, where growth laws are optimized to control size distribution width, because maintaining a uniform cell size prevents metabolic asynchrony and suppresses population oscillations, maximizing bioreactor stability and product yields [120].

Besides the growth law, PBE models ask to define how the division process is regulated—i.e., how the size/mass/age at which cells divide is controlled ($\gamma \left(x\right)$), and how volume and contents are partitioned at mitosis ($p\left(x,\;x^{\prime }\right)$).

Cell-size homeostasis is commonly described by 3 alternative but potentially coexisting modes of regulation: In a sizer model, the cell monitors its size and division is delayed until a threshold size is reached [121]; in an adder, the cell divides after adding a fixed volume increment to its birth size [91]; in a timer, the cell divides after a fixed growth period, irrespective of its size [122]. Experimentally, these control modes can be related to inferring $\gamma \left(x\right)$ from lineage-resolved measurements of size at birth, size at division, and inter-division times, which uncover how division propensity varies with size (or age). Such data are typically obtained through time-lapse quantitative microscopy in experimental setups that enable longitudinal single-cell tracking, including microfluidic devices and long-term live-cell imaging platforms [89–91]. Experimental and theoretical work has shown that real cells often display interpolations of these idealized behaviors and that different control strategies can coexist within the same population [92].

In case of a volume-structured PBE, a common choice is to represent a smooth sizer-like control—i.e., a division rate that is strongly peaked around a preferred division volume. For example, one may adopt a gaussian model

$$\gamma \left(V\right)=\gamma_{0}\text{exp}\left[-\beta \;{\left(V-V_{d}\right)}^{2}\right]$$ (25)

where *V* denotes the structuring size variable volume, $V_{d}$ is a preferred division size, $\gamma_{0}$ is a characteristic division rate, and *β* controls how sharply division is concentrated around $V_{d}$, as in [123,124]. Note that cells of the same lineage do not necessarily divide at the exact same size: Variability in size at division (“sloppy” size control) can be represented by prescribing a size-dependent division rate as in [125].

In general, size-dependent division timing is strongly altered by drugs that reshape $\gamma \left(x\right)$ without directly affecting growth rates [126]. In clinical modeling, Hinow et al. [127] parameterized this drug-induced alteration of *γ* using in vitro data to validate the PBE’s capacity to predict tumor growth. Moving from prediction to optimal control applications, Billy et al. [128] embedded a similarly calibrated $\gamma \left(x\right)$ into a size-structured PBE model to compute drug-delivery schedules that maximize tumor clearance while minimizing toxicity.

Beyond simple size-controlled division, timer- or adder-like behavior would generally require additional structure (e.g., age or added volume) beyond a single size coordinate. For instance, instead of using cell size as structural variable, one could adopt timer-like descriptions in which a cell-cycle “clock” variable is reset at each division event [129]. In this case, the division rate γ becomes a function of clock value, in line with classical clock-based models of the cell cycle [33,130]. However, such a clock—which implies approximately fixed interdivision times—is considered unrealistic because stochastic fluctuations in division timing cause the size at division to perform an effective random walk [92,114], and thus do not recapitulate division size homeostasis [90,91]. Therefore, timer-like descriptions that aim to remain realistic typically represent division by an age-dependent rate function γ chosen to reproduce the empirical distribution of interdivision times—with division size homeostasis emerging statistically [92,114,131,132].

As a minimal addition of mechanistic cell-cycle information, γ could in principle be modulated not only by physiological variables but also using coarse-grained molecular markers reflecting cell-cycle regulation, for instance, by using threshold-based readouts of cyclin–CDK activity (or related regulators) to gate commitment to division. Single-cell studies have quantified such CDK activity thresholds and could therefore provide natural ingredients to parameterize state-dependent transition or division rates [133,134].

Eventually, one has to define the rule by which a cell’s volume and contents are partitioned between daughter cells at division. This can be informed by mother–daughter-resolved measurements—for example, from time-lapse quantitative microscopy or other lineage-tracking approaches—that quantify daughter size, mass, or molecular content as a function of the mother’s pre-division state [135,136]. The partitioning rule is encoded in Eq. 17 by the kernel $p\left(x,\;x^{\prime }\right)$—which specifies the distribution of daughter sizes produced by a mother of size $x^{\prime }$. Conservation of volume at division is enforced by choosing $p\left(x,\;x^{\prime }\right)$ so that daughter sizes always sum to the mother’s size. Symmetric division corresponds to $p\left(x,\;x^{\prime }\right)=\delta \left(x-x^{\prime }/2\right)$, whereas broader or biased kernels allow unequal partitioning [135–138]. A common probabilistic kernel to model noisy symmetric division assumes a Gaussian distribution centered at half the mother’s size:

$$\begin{array}{c} p\left(x,\;x^{\prime }\right)=\frac{1}{\sqrt{2{\pi \sigma }^{2}x^{\prime }}}\text{exp}\left[-\frac{{\left(x-x^{\prime }/2\right)}^{2}}{2\sigma^{2}x^{\prime }}\right] \end{array}$$ (26)

where the variance scales with the mother size to reflect size-dependent partitioning noise [97,118]. Some asymmetric partitioning schemes use a binomial distribution: This is particularly appropriate for non-DNA molecules present in small numbers, where partitioning noise arises from random allocation events at mitosis [138]. Interestingly, clustered segregation that stems from vesicle packaging can be described as a multinomial process [135].

The choice of the partitioning kernel allows one to learn how mother–daughter inheritance rules shape the observable population distribution and its dynamics. Theoretical studies have shown that this kernel dictates the asymptotic population profile and long-time growth rate [139,140]; Michel [139] used a PBE framework to explain how cells can adapt their division asymmetry as an evolutionary strategy to maximize the overall population growth rate when nutrients are scarce, while Bernard and Gabriel [140] showed that when cells divide into 2 perfectly identical daughters, the population naturally tends to synchronize, explaining why researchers often observe persistent cyclic oscillations in flow-cytometry profiles rather than a stable, flat distribution.

**Fig. 4.**
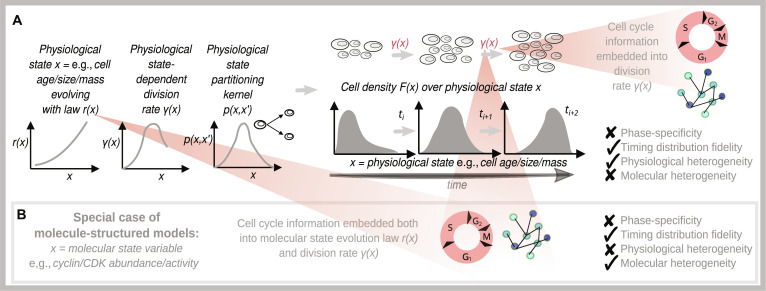
Continuously structured population models. (A) Physiologically structured models: The cell population is described by a continuous physiological state *x* (e.g., mass/size/age) evolving under a drift *r*(*x*), with division governed by a state-dependent rate $\gamma \left(x\right)$ and daughter-state inheritance encoded by a partitioning kernel $p\left(x|x^{\prime }\right)$. The population is represented by a cell density $F\left(x,\;t\right)$ transported in time along the physiological coordinate *x*. Cell-cycle information can enter these models through the division rate $\gamma \left(x\right)$, as indicated by the pink shaded inset. These models lack phase specificity but can generate faithful proliferation-timing distributions and resolve physiological, but not molecular, heterogeneity (bottom-right checklist). (B) Molecule-structured models: As a special case, the structuring coordinate *x* can represent a molecular variable (e.g., cyclin/CDK abundance or activity). Cell-cycle information can enter these models through the molecular evolution law *r*(*x*) and through the division rate $\gamma \left(x\right)$, as indicated by the pink shaded insets. These models lack phase specificity but can generate faithful proliferation-timing distributions and resolve molecular heterogeneity (bottom-right checklist).

So far, molecular regulation still enters PBE models solely through optional mechanistic rate closure, and the population model does not explicitly track molecular heterogeneity across the population. If desired, molecular variables can be promoted to structuring variables so that they evolve through the growth law *r*(*x*) and are inherited across generations through the partitioning kernel $p\left(x,\;x^{\prime }\right)$—moving PBE models beyond generic physiological heterogeneity (as reported by the checklist in Fig. 4B). Moreover, if model rate functions—e.g., the division rate γ—depend on the molecular coordinate, i.e., if they are mechanistically coupled to molecular regulation, inherited differences in molecular content feed back onto the future proliferative fate of daughter cells, creating an implicit coupling between molecular inheritance and population renewal. For instance, molecule-structured models that track cyclin content—which evolves according to the drift *r*(*x*) and determines the division propensity $\gamma \left(x\right)$—make it possible to analyze how such a coupling between cyclin and proliferation shapes long-term population dynamics [141–143]: Disruptions in single-cell cell-cycle machinery—such as reduced cyclin synthesis rates or delayed protein degradation—are propagated to the population level by shifting cells from an asynchronous replication regime to a collective, synchronized behavior. This explains the spontaneous emergence of coordinated division waves often observed in experimental flow-cytometry profiles.

Note that choosing a single structuring molecule *x* does not, by itself, embed the full regulatory network into the drift *r*. In practice, to circumvent prohibitive computational costs due to multidimensionality, detailed models are incorporated either offline—by projecting full network simulations onto the chosen coordinate to extract an empirical effective drift *r*(*x*) fed to the PBE—or online, via analytical model reduction to embed a simplified low-dimensional dynamical network directly.

Nevertheless, the population state can be structured along multiple molecular coordinates at once. Domschke et al. developed a spatiotemporal structured population framework in which cell density is structured by a vector of internal molecular binding states (e.g., multiple receptor–ligand binding variables emerging from a dynamical system) so that several molecular species are propagated jointly across the population and modulate the division rate γ in a continuous fashion (matching the strategy summarized in Fig. 6C) [144].

However, mechanistically informed closures relying on full CDK networks remain less common within single-compartment PBE frameworks, where the lack of explicit phase structure restricts the capacity to resolve phase-specific effects.

Once growth, division, and partitioning terms are specified, the PBE model predicts the time evolution of the population density $F\left(x,\;t\right)$ over the chosen physiological coordinate *x*. Depending on the choice of *x*, $F\left(x,\;t\right)$ can be constrained by repeated distributional measurements over the population, without necessarily tracking individual cells (see Table 1). For size- or volume-structured models, Coulter-counter measurements or, more approximately, flow-cytometry forward scatter provide snapshot or time-resolved size distributions [145]; interestingly, such volume/size distributions have been used in inverse formulations to infer model terms such as division rates [146]. For mass-structured models, suspended microchannel resonators (SMRs) can measure buoyant-mass distributions, whereas quantitative phase imaging/microscopy (QPI/QPM) can provide dry-mass distributions [108,147]. For age-structured models, lineage-resolved time-lapse microscopy can reconstruct cell-age distributions by measuring time since birth or last division [89,90]. Such age information has been used, for instance, to parameterize age-structured PBE models of therapy response [87]. For molecule-structured models, distributions over molecular abundance or activity can be constrained by snapshot measurements such as flow cytometry, quantitative immunofluorescence, fluorescent molecular reporters, or single-cell transcriptomic/RNA-FISH readouts, depending on the molecule considered. Relatedly, flow-cytometry fluorescence histograms have been used to infer proliferation-related parameters of molecule-structured PBEs from distributional time-series data [148].

In sum, continuously structured models provide a way to propagate physiological or molecular cell-to-cell heterogeneity and assess how it shapes population dynamics. Figure 4 outlines a simple single-compartment population-balance model with a physiological/molecular structuring variable, indicates where cell-cycle regulation can be incorporated, and delineates which behaviors such a model can or cannot capture: These models possess a within-state progression memory and they can faithfully reproduce cell-cycle timing distributions; they propagate cell-to-cell heterogeneity at the level of physiological or molecular variables of interest; however, because these basic PBE models lack explicit cell-cycle phase structure, they cannot resolve phase-specific effects. Early chemically structured population-balance models, such as those revisited by Fredrickson [149], already pointed to the need to explicitly represent cell-cycle phase progression within otherwise continuous physiological state descriptions, motivating hybrid formulations that couple continuous physiological coordinates with explicit cell-cycle phase compartments.

### Structured phase-compartment population-balance models

Along with any structuring coordinates of interest, these population-balance models explicitly represent cell-cycle stage and thus assign to each phase *p* a cell density $F_{p}$. For simplicity, we restrict here to the case in which this density depends on a single within-phase structuring variable *x* so that $F_{p}\left(x,\;t\right)$ denotes the density of cells in phase *p* with state *x* at time *t*. The variable *x* may represent size/mass, age-in-phase (i.e., time since entry into phase *p*), or another physiological coordinate; however, PBE frameworks with phase compartments align particularly well with introducing a phase-specific age variable *x*, which tracks how long cells have spent in a given phase. Unlike purely discrete compartmental models, such a description captures not only how many cells occupy a phase but also how far they have progressed within it. This leads to formulations where within-phase transport is coupled to age-dependent transition rates between phases, such as [131,150–152].

Since structuring by size or mass follows the same principles outlined in the previous section when applied to phase compartments, we focus here on phase age as the central coordinate for phase-resolved formulations. Quiescent-proliferative partitions follow a conceptually analogous, albeit structurally simpler, formulation; we therefore do not discuss them explicitly here in their general form, but we will introduce specific instances as concrete examples of compartmentalized population-balance models.

Let P denote the set of cell-cycle phases (e.g., $P=\left\{G1,S,G2,M\right\}$). For each phase p∈P, let $F_{p}\left(x,t\right)$ denote the density of cells in phase *p* at internal age *x*, i.e., time since entry into that phase, at time *t*. Since *x* is a time-like coordinate, it increases deterministically at unit speed within each phase so that its drift in phase *p* is

$$r_{p}\left(x\right)=\frac{\mathrm{dx}}{\mathrm{dt}}=1.$$ (27)

Cells leave each phase *p* (entering the next, *p*′) according to an age-dependent rate $\gamma_{p\to p^{\prime }}\left(x\right)$. Thus, the generic sink term that encodes division in the basic PBE [Eq. (18)] is replaced by a phase-exit term $-\gamma_{p\to p^{\prime }}\left(x\right)\;F_{p}\left(x,\;t\right)$, which removes cells from phase *p* as they transition to the next phase. Thus, in analogy with Eq. (18), the balance for each phase *p* can be written in flux form as

$$\begin{array}{c} \frac{\partial F_{p}\left(x,\;t\right)}{\partial t}+\frac{\partial }{\partial x}\;\left[F_{p}\left(x,\;t\right)\right]=-\gamma_{p\to p^{\prime }}\left(x\right)\;F_{p}\left(x,\;t\right). \end{array}$$ (28)

Note that, according to Eq. (17), a phase-specific kernel $p_{p}\left(x|x'\right)$ is expected to specify how cells leaving a phase *p* with state $x^{\prime }$ are reintroduced into the next phase $p^{\prime }$ with coordinate *x*. However, as the structuring coordinate is the phase-age, it is reset to *x* = 0 at each transition. As a result, the kernel term reduces to a boundary reinjection rule: The outflow from phase *p* is routed into the inflow of phase $p^{\prime }$ at the boundary *x* = 0:

$$F_{p^{\prime }}\left(0,\;t\right)=\int_{0}^{\infty }\gamma_{p\to p'}\left(x\right)\;F_{p}\left(x,\;t\right)\;\mathrm{dx},$$ (29)

where the integral runs over all phase-*p* ages $x\in \left[0,\infty \right)$.

Note that the *p*-to-$p^{\prime }$ phase transition corresponds to mitosis (p=M, $p^{\prime }=G1$), each exiting mother cell produces 2 daughters entering $G_{1}$, so the same routing condition acquires a factor 2:

$$\begin{array}{c} F_{G1}\left(0,\;t\right)=2\int_{0}^{\infty }\gamma_{M\to G1}\left(x\right)\;F_{M}\left(x,\;t\right)\;\mathrm{dx}. \end{array}$$ (30)

One can allow an external perturbation/input *u*(*t*) (e.g., growth factors or drug treatment) to modulate age-dependent phase-transition rates directly, i.e.,

$$\gamma_{p\to p^{\prime }}=\gamma_{p\to p^{\prime }}\left(x;\;u\left(t\right)\right).$$ (31)

In this way, with *x* providing an internal clock for each stage, the model makes it possible to capture phase-specific delays, arrests, and synchronization. For instance, antimitotic perturbations selectively trigger the spindle-assembly checkpoint to trap cells in mitosis, inducing highly heterogeneous delays that depend directly on the time already spent in-phase [153].

This makes phase-age-structured frameworks natural for optimizing perturbation timing to control phase residence and synchronization, which ultimately impact population growth. Billy et al. [154] used this framework to model competing tumor and healthy tissue populations under chronotherapeutic protocols. They analyzed how the time-dependent pharmacological blockade of phase transitions (i.e., through $\gamma_{p\to p^{\prime }}$) can be scheduled to maximize tumor degradation while respecting toxicity constraints in healthy cells. Later formulations extended this approach to optimize time-scheduled drug delivery by embedding explicit PK/PD modules that translate clinical intravenous infusion profiles into time-varying phase-transition rates to minimize systemic toxicity [155].

From an experimental standpoint, adding explicit phase compartments to continuously structured models introduces phase-specific terms that bulk readouts usually constrain only as combinations, failing to ensure model identifiability. Chaffey et al. [156] illustrated this issue by fitting an age-structured population-balance model with an explicit $G_{1}\to S$ checkpoint to experimental time-course data; they showed that distinct transition-rate functions can reproduce the same population-level observations yet imply markedly different internal phase distributions. On the other hand, phase-resolved abundance readouts alone neglect the distribution of the structuring variable within each phase, and therefore cannot identify how transition rates depend on that coordinate. Effectively, phase-resolved PBE frameworks require combining distributional data of the structuring variable with readouts that map directly to the internal phase architecture of the cycle (see Table 1). Single-cell imaging with FUCCI phase reporters satisfies this need by tracking how much time individual cells spend in a given phase. For example, Billy et al. [131] used FUCCI fluorescence trajectories to model the $G_{1}\to S$ transition under varying growth-factor conditions. This allowed them to uniquely identify how growth factors modulate age-dependent transition rates to synchronize the population. Basse and Ubezio [157] formulated an age-in-phase-structured model in which phase-transition functions are calibrated against DNA-content flow-cytometry profiles of tumor-cell populations under unperturbed growth and anticancer therapies. This enabled DNA-content profiles to be translated into altered transition terms so that the model could reproduce therapy-driven accumulation or depletion of cells in specific cell-cycle phases.

While not originally intended to calibrate PBEs, some studies provide experimental readouts that can inform these models: Pulse-labeling data capture phase completion times and their cell-to-cell variability [158]; in case of mass/size-structured phase-compartment PBEs, measuring these features alongside cell-cycle position reveals how physical distributions evolve across different cycle stages [126,159].

Beyond empirical calibration, PBE phase-transition rates can directly embed the underlying cell-cycle biology—making it possible to interpret phase-specific population effects through a regulatory lens, or make new predictions.

A first level of mechanistic enrichment is to allow phase exit to depend on a coarse-grained regulatory marker *z*(*t*)—e.g., cyclin/CDK activity or checkpoint status—despite not accounting for a full dynamic network, i.e.,

$$\gamma_{p\to p^{\prime }}=\gamma_{p\to p^{\prime }}\left(x,\;z\left(t\right)\right),$$ (32)

For a full mechanistic coupling, as usual *z*(*t*) is generated by an intracellular dynamical system and optionally driven by external inputs *u* (e.g., growth factors or drugs):

$$\frac{\mathrm{dz}}{\mathrm{dt}}=C\left(z;\;u\right),$$ (33)

affecting $\gamma_{p\to p^{\prime }}\left(x,\;z\left(t\right)\right)$ and thereby making phase transitions emergent from intracellular dynamics rather than imposed jumps.

For example, Hodgkinson et al. [160] formulated a cell-cycle-structured PBE model where transition rates depend continuously on temporal cyclin levels generated by the biochemical loop of p53, Mdm2, and mitotic cyclins (mapping to the strategy schematized in Fig. 6C). By coupling the $G_{2}\to M$ transition rate (i.e., $\gamma_{G_{2}\to M}$) to such loop, they computationally reproduced the well-known cell-cycle arrest in response to DNA damage that allows for repair. Batool and Bajcinca [161] implemented a similar strategy in a quiescent-proliferative age model by coupling Q/P transition terms to an intracellular network centered on the cyclin D/CDK4-6–Rb–E2F axis. Here, the dynamical network state modulates the quiescence-to-proliferation switching with a continuous strategy (Fig. 6C) under growth-factor input. This enabled to analyze how cell-to-cell heterogeneity in the molecular components of the Rb–E2F pathway dictates both the fraction of cells that successfully re-enter the proliferative cycle and the delay in population-level growth.

A closely related mechanistic closure is provided by Pisu et al. [129]. However, this approach differs from the embeddings above in that the regulatory coupling is performed “offline”, i.e., by computing a static phase duration value and converting it into a fixed transition rate—thus mapping to the strategy in Fig. 6A. Here, intracellular regulation is described by a dynamical system $\frac{\mathrm{dz}}{\mathrm{dt}}=C\left(z;\;u\right)$, where *z* represents the state of a cyclin–CDK network of the type developed in [162–165], and *u* represents external cues such as mitogenic, stress, or pharmacological signals. Such molecular module is first simulated independently to extract effective phase durations $\tau_{p}$, defined by threshold-crossing events along the molecular trajectory *z*(*t*) (e.g., S-phase entry upon CDK2 activity exceeding a critical level); then, $\tau_{p}$ is fed to the population model by defining an internal phase clock $\xi_{p}$—defined as a normalized phase-age variable $\xi_{p}=x/\tau_{p}$ taking values in $\left[0,1\right]$—evolving as

$$\frac{d\xi_{p}}{\mathrm{dt}}=\frac{1}{\tau_{p}}$$ (34)

and with the phase transition occurring deterministically when $\xi_{p}$ equals 1. This offline mode is particularly useful when comparing a finite set of predefined conditions, such as different doses or phenotypes: The intracellular module can be simulated separately for each condition, and then the corresponding phase durations $\tau_{p}$ can be extracted and used in the PBE model. This enabled to simulate batch mammalian cultures under cell-cycle perturbations, using PBEs with both cell volume and internal phase clock structure [166].

**Fig. 5.**
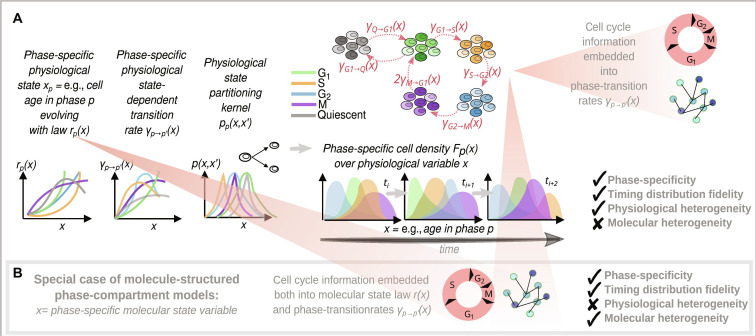
Continuously structured phase-compartment population models. (A) Physiologically structured phase-compartment models: The cell population is subdivided into discrete cell-cycle phases *p*, and each phase is described by a continuous physiological state x (e.g., mass/size/age) evolving under a phase-specific drift $r_{p}\left(x\right)$, with phase progression governed by a state-dependent transition rate $\gamma_{p\to p^{\prime }}\left(x\right)$ and daughter-state inheritance encoded by a phase-specific partitioning kernel $p_{p}\left(x|x^{\prime }\right)$. The population is represented by phase-specific cell densities $F_{p}\left(x,\;t\right)$ transported in time along the physiological coordinate x. Cell-cycle information can enter these models through the phase-transition rates $\gamma_{p\to p^{\prime }}\left(x\right)$, as indicated by the pink shaded inset. These models resolve phase specificity, can generate faithful phase-timing distributions, and resolve physiological, but not molecular, heterogeneity (bottom-right checklist). (B) Molecule-structured phase-compartment models: As a special case, the phase-specific structuring coordinate x can represent a molecular variable (e.g., cyclin/CDK abundance or activity). Cell-cycle information can enter these models through both the molecular evolution law $r_{p}\left(x\right)$ and through the phase-transition rates $\gamma_{p\to p^{\prime }}\left(x\right)$, as indicated by the pink shaded insets. These models resolve phase specificity, can generate faithful phase-timing distributions, and resolve molecular heterogeneity (bottom-right checklist).

Figure 5A presents a general phase-compartment population-balance approach, indicates where cell-cycle regulatory mechanisms can be incorporated, and clarifies which behaviors these models can or cannot capture. As indicated in the checklist, this formulation combines phase specificity and physiological cell heterogeneity with phase progression memory that can recapitulate phase timing distributions. Still, these models do not propagate molecular heterogeneity unless explicit molecular structure is introduced.

Molecular structuring coordinates become essential in phase-compartment PBE models whenever molecular variability dictates cell-cycle phase-specific effects, e.g., when investigating how the asymmetric inheritance of proteins and drugs dictates checkpoint kinetics and generates divergent subpopulation fates. In such cases, cell-cycle regulators can be promoted to structural variables and made phase-specific—capturing distinct regulators and checkpoints within $G_{1}$, *S*, $G_{2}$, and M—rather than acting as a single generic division marker as in a pooled single-compartment formulation. This corresponds to mapping the formulation of Eq. (28) by replacing the phase-age coordinate *x* with the molecular state. Here, $F_{p}\left(x,\;t\right)$ represents the density of cells in phase *p* with molecular state *x* (for instance, cyclin abundance or a proxy for CDK activity); $r_{p}\left(x\right)$ describes the biochemical drift—i.e., the evolution of the molecular state within phase *p* driven by synthesis, degradation, and activation dynamics; and $\gamma_{p\to p^{\prime }}\left(x\right)$ represents a molecular-state-dependent exit rate from phase *p* (or division, when p=M). Molecular content is mapped at each phase transition using the kernel $p_{p}\left(x|x^{\prime }\;\right)$, which specifies how the molecular state is reinjected at $p\to p^{\prime }$ entry. The total inflow into the subsequent phase $p^{\prime }$ at molecular state $x_{0}$ is given by the boundary condition integrating over all exiting molecular states of phase *p*:

$$\begin{array}{c} F_{p^{\prime }}\left(x_{0},\;t\right)=\int p_{p}\left(x_{0}|x\right)\;\gamma_{p\to p^{\prime }}\left(x\right)\;F_{p}\left(x,\;t\right)\;\mathrm{dx}, \end{array}$$ (35)

In particular, at mitosis (p=M, $p^{\prime }=G1$) molecular content is partitioned between daughter cells according to:

$$\begin{array}{c} F_{G1}\left(x_{0},\;t\right)=2\int p_{M}\left(x_{0}|x\right)\;\gamma_{M\to G1}\left(x\right)\;F_{M}\left(x,\;t\right)\;\mathrm{dx}. \end{array}$$ (36)

where the factor 2 encodes the stoichiometry of division.

In this framework, experimental readouts that inform PBE model terms must concurrently map both the molecular coordinate and the specific phase compartment: Single-cell assays are needed to track *x* and assign *p* simultaneously (see Table 1). In live-cell setups, the current phase is identified either by coexpressing a cell-cycle indicator (such as FUCCI) alongside a reporter for the molecule of interest or by exploiting the characteristic temporal profile of the molecule itself (e.g., the abrupt drop in CDK activity that signals mitosis). Tracking these biosensors over time yields phase-segmented single-cell trajectories, which can directly quantify both the phase-specific biochemical drift, $r_{p}\left(x\right)=\frac{\mathrm{dx}}{\mathrm{dt}}$, and the corresponding gating rate, $\gamma_{p\to p^{\prime }}\left(x\right)$. Cappell et al. [167] tracked CDK2 activity and geminin accumulation to segment single-cell trajectories into explicit phase compartments ($p\in \left\{G_{1},\;S,\;G_{2},\;M\right\}$), showing that the $G_{1}\to S$ transition is gated by a specific molecular threshold—the inactivation of APC/C. Such live-imaging data provide suitable ingredients to parameterize a phase-resolved molecule-structured PBE; e.g., in a given phase *p*, the empirical time derivative of the biosensor—corresponding to dx/dt—directly resolves the phase-specific drift $r_{p}\left(x\right)$, while its distribution at the transition point can define the molecule-dependent transition rate $\gamma_{p\to p^{\prime }}\left(x\right)$.

When only static snapshot assays (e.g., immunofluorescence) are available, cell-cycle phases can be resolved via costaining (e.g., DNA dyes), while single-cell measurements of a regulatory molecule yield its distribution $F_{p}\left(x,\;t\right)$ within each phase. Note that classical bulk assays (e.g., EdU/BrdU pulse labeling) only provide the total phase fractions—mathematically corresponding to the phase-integral $\int F_{p}\left(x,\;t\right)dx$—thereby losing all single-cell molecular heterogeneity. These phase-specific static distributions of *x* could potentially serve to infer PBE drift and/or gating terms from molecular snapshots, which remains a largely open challenge in the PBE framework.

When checkpoint-specific mechanisms are resolved, molecule-structured phase-compartment formulations can be coupled directly to cell-cycle regulation through dynamic variables or intracellular modules (Fig. 5B). In this setting, mechanistic information enters both through the drift term $r_{p}\left(x\right)$—which contains the phase-specific regulatory dynamics—and through the molecule-dependent phase-transition rate $\gamma_{p\to p'}\left(x\right)$. For instance, a spindle-assembly checkpoint model informing mitotic exit [76] can be naturally embedded by propagating the relevant mitotic coordinate within that phase to dictate the corresponding exit propensity $\gamma_{M\to G1}\left(x\right)$.

**Fig. 6.**
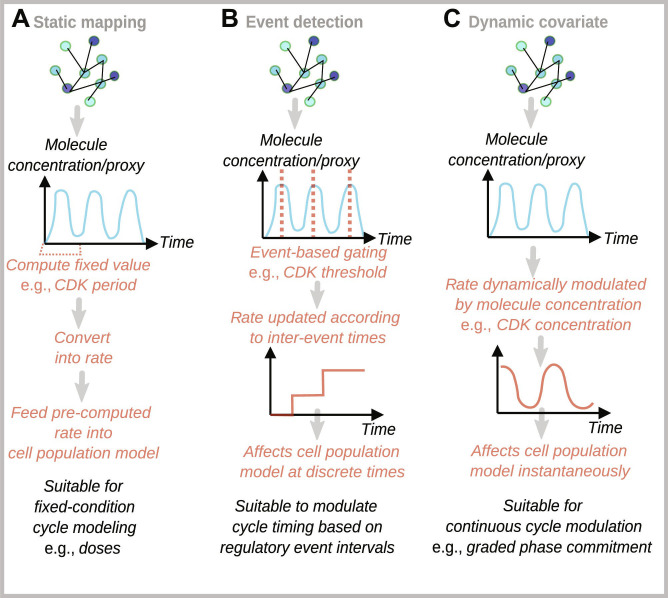
Mechanistic embedding strategies for coupling intracellular regulation to cell-cycle-informed population models. (A) Static mapping: Intracellular dynamics are simulated offline, and their extracted features (e.g., CDK activity period) are subsequently mapped onto population-level rates. (B) Event-based embedding: Regulatory events (e.g., threshold crossings) are detected from intracellular dynamics to extract inter-event times and update rates at discrete times within the population model. (C) Dynamic covariate embedding: Molecular state variables or proxies continuously modulate population model rates.

A relevant example by Bekkal Brikci et al. [151] integrated the intracellular cyclin–CDK regulatory network directly into the compartment-specific drift term $r_{p}\left(x\right)$ of a quiescent-proliferative PBE model; moreover, they modeled a molecule-dependent propensity to remain quiescent or to (re-)enter sustained cycling through a gating function $\gamma_{p\to p^{\prime }}\left(x\right)$ continuously modulated by cyclin–CDK levels (mapping to Fig. 6C). This enabled them to interpret well-known population-level behaviors—such as the transition from tissue homeostasis to unlimited tumor growth—as emergent directly from the interplay between molecular feedback loops and transition propensities [151].

In sum, phase-structured PBEs provide the most expressive deterministic framework reviewed here, because they combine phase specificity, faithful within-state progression timing, and physiological or molecular heterogeneity.

## Discussion

Deterministic models of cell population dynamics have evolved substantially over the last century, spanning phenomenological growth laws, compartment-based formulations, and physiologically structured population-balance frameworks. Yet, considering that population-level expansion ultimately emerges from cell-cycle progression, a unified landscape of how population dynamics can be coupled to cell-cycle mechanisms is still lacking. Indeed, modeling efforts have historically focused on refining specific architectures within isolated subfields—e.g., tumor-growth modeling or bioprocess engineering, and some recent reviews have provided overviews of modeling approaches and their applications in specific biological contexts [168].

**Fig. 7.**
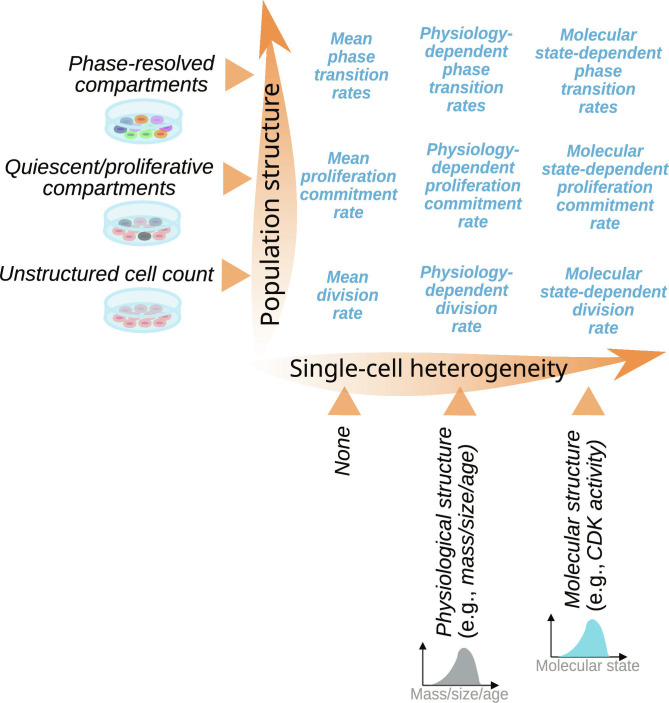
Conceptual map of how cell-cycle information enters deterministic population models. The vertical axis indicates increasing discrete population structure, from unstructured cell counts to quiescent/proliferative and phase-resolved compartments. The horizontal axis indicates increasing continuous single-cell heterogeneity, from no resolved single-cell coordinate to physiological or molecular state structure. Each region shows in which form cell-cycle information can be incorporated at that level of resolution.

By organizing modeling approaches according to cell population structure and the mechanistic specification of division and cell-cycle phase transitions, we aimed to clarify what each level of model detail can represent, what data it matches, what features it necessarily leaves unresolved, and how molecular mechanisms can be embedded. Although the 2 dimensions of population structure and cell-cycle mechanism are conceptually independent—and thus mechanistic closures can, in principle, be embedded across structural levels—in practice more detailed biochemical descriptions of cell-cycle regulation align most naturally with population states that expose cell-cycle heterogeneity, such as checkpoints or phase transitions, and are otherwise absorbed into coarse effective terms. This creates a correspondence between population-level resolution and the mechanistic detail that can be supported. Figure 7 summarizes how cell-cycle regulatory information is coarse-grained across models differing in population structure and single-cell heterogeneity.

Moreover, the data themselves impose an intrinsic limit on mechanistic enrichment: When observations lack sufficient phase or regulatory resolution, distinct descriptions collapse onto the same observable population dynamics. Nevertheless, the rapid expansion of single-cell technologies now reshapes what cell-cycle-driven population models can realistically constrain, especially at the molecular level [169]. Time-resolved fluorescent reporters and lineage tracking already allow estimation of dwell-time distributions and checkpoint windows. Beyond these, high-throughput single-cell transcriptomic and multi-omic measurements provide unprecedented access to regulatory heterogeneity across large populations.

A natural direction is therefore to move beyond a scalar modulation of population dynamics terms and to embed at least low-dimensional regulatory coordinates directly into population formulations. In particular, molecule-structured population-balance frameworks provide a scaffold to propagate heterogeneous molecular states rather than compress them into effective rate modifiers.

When fully specified intracellular ODE networks are unavailable or non-identifiable, low-dimensional regulatory coordinates may be inferred directly from single-cell data—e.g., principal regulatory axes, pseudotime trajectories, or manifold embeddings—and treated as effective dynamical variables whose drift captures the coarse evolution of intracellular regulatory state [170–172].

Although still operating at the level of static phenotype identification rather than dynamical reconstruction, recent single-cell RNA-seq-based computational studies of tumor heterogeneity and treatment-associated resistant subpopulations, such as [173,174], provide examples of how single-cell molecular snapshots can identify phenotypic or regulatory states, such as treatment-associated resistant subpopulations. In a population-dynamical model, these states could then be represented either as discrete phenotypic compartments or as continuous molecular/regulatory coordinates whose abundance or distribution changes under disease progression or perturbation. The same kind of data can be used to infer temporal direction with techniques such as RNA velocity, yielding an empirical estimate of a drift field over the state space that might directly parameterize population-dynamical models [175,176].

More generally, when explicit mechanistic networks are lacking, such a data-driven drift inference can be embedded within hybrid formulations: The intracellular drift field can be learned directly from data (e.g., via neural ODE parameterizations), while the population-level evolution remains governed by a structured transport equation [177,178].

Integration with lineage-tracing data (e.g., live-cell imaging lineage tracking or lineage barcoding) represents another promising direction for what concerns inheritance and regulatory memory across generations: It could enable direct inference of inheritance kernels, constraining cell partition functions and molecular state propagation in a way not accessible from snapshot data alone. This may bring structured deterministic models closer to experimentally measurable genealogical dynamics while retaining their mean-field formulation.

When lineage-resolved data also report differentiation events, they would naturally motivate extensions that account for the generation of multiple phenotypic lineages. Although this review has focused on population dynamics driven by division and loss, deterministic population formulations can accommodate lineage branching through coupled population compartments representing distinct differentiation stages [152,179,180].

On the other hand, a largely missing counterpart is a mirror framework for cell loss: Population dynamics models, in which death (and irreversible loss of cycling capacity) is coupled mechanistically to intracellular apoptotic programs, rather than treated as a purely phenomenological aspect, are still poorly explored [181]. Such an extension would complete the “other side of the coin” relative to cycle-informed population models.

As dimensionality grows—for instance, when tracking phase compartments with multiple physiological or molecular markers—computational feasibility and identifiability become central concerns. PBEs can rapidly become high-dimensional and demanding to formulate, parameterize, and solve [97,182]. A variety of numerical strategies have been developed to mitigate this burden, including moment-closure approximations, sectional and discretization methods, and stochastic Monte Carlo solvers for PBEs [182–184]. Spectral methods have also been explored to mitigate dimensionality by projecting multi-variable PBEs onto reduced functional bases [185].

Nowadays, operator-learning approaches offer another potential workaround: Neural operators can be trained to rapidly approximate PBE solutions for new inputs, while physics-informed variants keep the learned solutions consistent with the underlying transport and renewal equations. This can accelerate simulations without abandoning the governing model [186,187].

In parallel, Bayesian data-assimilation frameworks treat unknown model terms as latent functions to be inferred from heterogeneous observations—such as growth curves, phase fractions, and molecular readouts [188,189].

Nevertheless, these extensions must be approached cautiously. Snapshot single-cell omics do not provide true temporal trajectories and require careful dynamical interpretation; expression levels are imperfect proxies for biochemical activity; and increasing molecular resolution without commensurate data support risks overparameterization and loss of interpretability. The appropriate degree of model resolution therefore remains context-dependent.

Finally, some biological questions concern stochastic realizations of cell genealogies rather than the evolution of population-level state distributions. This is the case when explicit single-cell trajectories, lineage histories, or stochastic fate realizations are themselves the object of study, and therefore require individual-based formulations. Structured population models, instead, propagate heterogeneity through deterministic equations for cell-state densities without tracking individual cells. Nevertheless, many agent-based and stochastic individual-level formulations can be related to structured PBEs through mean-field or kinetic limits, providing a theoretical connection between individual-based simulations and the deterministic population-level equations reviewed here [190–192].

## Conclusion

The existing body of deterministic models provides a solid foundation for the emergence of a more integrated framework of cell-cycle-informed population dynamics.

As computational tools mature and single-cell datasets gain temporal and molecular depth, the most impactful developments are likely to emerge from principled combinations of physiological or molecular population structure and cell-cycle mechanism embeddings, selected according to the biological question and constrained by the data needed to preserve identifiability.
